# “We Listen to Women's Experiences of Intimate Partner Violence, but We Stop Them When It Comes to Sexual Violence:” Training Needs of Shelter Workers

**DOI:** 10.1177/10778012251347624

**Published:** 2025-06-16

**Authors:** Stéphanie Couture, Geneviève Brodeur, Mylène Fernet, Martine Hébert, Catherine Flynn, Louise Lafortune

**Affiliations:** 1 Department of Psychology, Université de Montréal, Montreal, Quebec, Canada; 2 Department of Sexology, 14845 Université du Québec à Montréal, Montreal, Quebec, Canada; 3 Department of Social Sciences and Humanities, 14661 Université du Québec à Chicoutimi, Chicoutimi, Quebec, Canada; 4 Regroupement des maisons pour femmes victimes de violence conjugale, Quebec, Canada

**Keywords:** intimate partner sexual violence, intimate partner violence shelters, needs assessment, qualitative analysis

## Abstract

The need for effective training to support survivors of intimate partner sexual violence (IPSV) remains underexplored. This study aimed to identify IPSV-related training needs among shelter workers. A comprehensive qualitative analysis was conducted with 179 shelter advocates through focus groups and a World Café. The analysis revealed four overarching needs: (a) enhancing ease; (b) clarifying the scope and limitations of interventions; (c) developing skills to respond effectively; and (d) guiding survivors through medico-legal and legal procedures. These findings provide key recommendations for improving the quality of support, enhancing survivors’ recognition of IPSV, and facilitating their access to justice and recovery.

Intimate partner sexual violence (IPSV) affects a substantial portion of the population, especially women. A recent meta-analysis found that, worldwide, one in 10 women (9.6%) have reported having experienced at least one form of IPSV in their lifetime (White et al., 2024). This proportion reached 33% when considering women using intimate partner violence shelter services (Moreau, 2019). IPSV is situated on a continuum of nonverbal behaviors and verbal pressures, including sexual coercion, cyber sexual violence, and sexual fondling. These behaviors are used to force or pressure an intimate partner (or ex-partner) into engaging in unwanted sexual activities (Bagwell-Gray et al., 2015). IPSV often leads to significant social stigma and privacy concerns, which explains why women who have experienced IPSV are less likely to seek informal support (e.g., from family and friends; Wright et al., 2022). However, women experiencing both physical intimate partner violence and IPSV are more likely to access formal support services than women reporting only psychological or physical violence (e.g., intimate partner violence shelters; Wright et al., 2022). Studies have revealed that women report other important barriers to help-seeking, including confusion about IPSV in intimate contexts, making it difficult to characterize this form of violence as abuse (Wright et al., 2022). The literature also highlighted various challenges faced by shelter advocates (i.e., individuals who work within or on behalf of intimate partner violence shelters; Dunn & Powell-Williams, 2007; Merchant & Whiting, 2015; Voth Schrag et al., 2022). However, to the best of our knowledge, only one study provided insights into specific training needs related to IPSV (Helps, 2024).

## Intimate Partner Violence Shelters and Shelter Advocates Challenges

Shelters are specialized in intervention for women experiencing all forms of intimate partner violence, including physical, psychological, and sexual violence. In general, shelter advocates describe their practice as being feminist, intersectional, and client-centered (Nichols, 2013). As part of their mandate, they are called upon to provide information about intimate partner violence and accompany women on their empowerment journey, while respecting their individual pace and decisions (Nichols, 2013).

The literature has provided evidence of the challenges encountered by shelter advocates when intervening with female survivors of intimate partner violence. A study revealed that when asked about the challenges they face, shelter advocates were more likely to point to challenges arising related to service users, with the most frequently mentioned challenge being women's decisions to either remain in or return to abusive relationships (Dunn & Powell-Williams, 2007). In a qualitative study of 19 shelter advocates, participants reported these challenges and many others, such as repeatedly hearing women's difficult stories, enforcing rules in the shelter, and accessing resources (e.g., limited shelter stays; Merchant & Whiting, 2015). Participants emphasized the key role of a supportive shelter culture in effectively addressing these challenges, thereby influencing advocate satisfaction and retention (Merchant & Whiting, 2015). Moreover, shelter advocates are at an increased risk for compassion fatigue (i.e., exhaustion from working with trauma survivors that affects workforce practices; Voth Schrag et al., 2022). While these studies are relevant to better understand the challenges faced by shelter advocates in their work with female survivors of IPSV, to our knowledge, there is currently no research investigating their specific challenges related to IPSV.

## Intimate Partner Sexual Violence and Challenges for Practice

IPSV is still an underexplored form of intimate partner violence, possibly attributed to its intersection vis-a-vis the fields of sexual violence and intimate partner violence (Bagwell-Gray et al., 2015). IPSV is perceived as less serious than sexual violence perpetrated by a nonpartner due to a lack of understanding of the dysfunctional dynamics related to sexual consent in violent relationships; it has been associated with increased symptoms of posttraumatic stress and depression, as well as a high number of deaths by suicide and homicide (Sanchez et al., 2023). This misapprehension has the potential to hinder survivor's ability to identify the abuse as sexual violence (Sanchez et al., 2023). Research has indicated that women often struggle to recognize and assess IPSV because they feel that they cannot refuse their partner's sexual advances, convince themselves that they are to blame for the sexual difficulties experienced in their relationship, and want to protect their partner's feelings (e.g., masculine fragility; Tarzia, 2021). This accentuates the potential existence of unique needs and challenges sparsely documented that intimate partner violence services, like intimate partner violence shelters, might not overtly address (Wright et al., 2022). This underscores the relevance of a more in-depth study of shelter advocates’ needs related to IPSV to ensure comprehensive support for female survivors. By acknowledging the nuanced aspects of IPSV, service providers could better tailor interventions to the complex dynamics and impacts of IPSV.

IPSV is a sensitive issue, and sexuality may be an uncomfortable topic to address (Bagwell-Gray et al., 2015), potentially leading to challenges in the practice of professionals working with female survivors. A recent study examined screening and risk assessment of IPSV in the context of perpetrator interventions among 97 practitioners (Helps, 2024). Findings revealed significant challenges in screening and risk assessment for IPSV, including a lack of communication and silos between services, experiences of shame as a barrier to disclosure, misconceptions of sexual consent and what is expected in intimate relationships, and a lack of assessment tools better suited for identifying IPSV that do not involve physical force (Helps, 2024). Although many participants had experience working with survivors, this study focused primarily on interventions with perpetrators using a survey (Helps, 2024). Consequently, the survey's results may not have fully captured the training needs for effectively supporting IPSV survivors. Some studies have documented challenges faced by service providers in their practice with adult survivors of sexual violence (Espinosa, 2023; Ullman, 2014); however, the particularities of abuse committed in a domestic context have not been highlighted. In a sample of 12 therapists, participants expressed emotional difficulties working with adult survivors due to similarities between their own experiences and those of service users (Ullman, 2014). In another qualitative study of mental health professionals working with female survivors of sexual assault, participants reported the need for therapeutic detachment and reflexivity, and the difficulty of remaining neutral and consolidating their own reactions (Espinosa, 2023). Examining the training needs of shelter advocates regarding IPSV could provide a thorough documentation of their specific challenges and inform the development of targeted training programs and initiatives to enhance service provision to female survivors of IPSV.

## Current Study

Notwithstanding their acknowledged expertise in the area of intimate partner violence, shelter workers, as reported by our partner organization which constitutes an extensive network of shelters*,* have expressed the necessity for training to effectively address cases involving female survivors of IPSV. This observation has emerged in a changing social context, with a shift in the demographic profile of women seeking help in intimate partner violence shelters, particularly a noticeable overrepresentation of women aged 25–34, immigrants, and those self-identifying as First Nations people (Moreau, 2019). In line with the principles of the participatory approaches, the research team has collaborated with a large organization to gather input from shelter advocates and incorporate moments of differing opinions to be discussed to negotiate differences and validate key findings (Guijt, 2014). Mobilizing a qualitative approach has provided in-depth insights into the challenges faced by shelter workers and has helped assess training targets that are most likely to improve the quality of services provided. The main goal of the current study is to identify shelter workers’ training needs to effectively address IPSV within intimate partner violence shelters.

## Method

The current qualitative study was conducted between May 2022 and May 2023 as part of a broader participatory action research involving shelter advocates and women receiving intimate partner violence shelter services. The analyses in this study specifically focus on shelter advocates’ viewpoints.

### Recruitment and Procedures

Recruitment was supported by our partner organization, which represents 46 intimate partner violence shelters across the province of Quebec. Data were collected in two consecutive phases via asynchronous online focus groups and an in-person World Café session.

#### Asynchronous Online Focus Group

Asynchronous online focus groups have gained popularity due to the widespread use of technology (Ybarra et al., 2014). This method uses chat tools for moderated and structured online discussions, allowing participants to engage at times that suit their schedules (Williams et al., 2012). It enhances flexibility in exchanges and participation, overcoming geographical barriers and individual characteristics (e.g., embarrassment, stigma) that may hinder certain voices from being heard (Williams et al., 2012). A total of four focus groups were held, including 18 shelter workers. First, the study advertisement was shared via email to all shelter workers employed in intimate partner violence shelters affiliated with the organization. To be eligible, shelter workers needed to be employed by one of the 46 shelter members of the organization and involved in providing services to female survivors of intimate partner violence at the time of the study. Shelter workers interested in the study were invited to complete a form to express their willingness to participate. The research team then contacted them by phone to confirm their eligibility based on the previously mentioned criteria, and to provide information about the procedures (e.g., duration of the focus group, installation protocol for Telegram). Among the 26 shelter workers who expressed interest, four did not respond when contacted, one did not have access to an electronic device to download Telegram (i.e., a free-of-charge application with multiple security settings and chat groups suitable for qualitative research), and three were awaiting the start of a group. The latter had expressed their interest after the completion of a group for which they were eligible, but the number of participants in their experience bracket was insufficient to form a new group. Participants were grouped based on their years of experience as shelter workers (i.e., less than 3 years, 3–7 years, and 7 years or more). A semistructured question grid was developed based on an outcome-focused systematic review of intimate partner violence education interventions for healthcare professionals (e.g., social workers, and nurses; Sawyer et al., 2016). The grid, which was used by research assistants to facilitate group dialogue, included a series of open-ended questions and subquestions designed to stimulate discussions about knowledge, attitudes, skills related to IPSV, actual challenges encountered in their interventions with IPSV survivors, and their perceived training needs. Participants were asked to answer these questions at their convenience (i.e., asynchronously) and discussed a total of four main questions. One question every 3 days, considering their overall workload, was asked via the Telegram application within groups consisting of four to six shelter workers. Focus group participants were asked to create pseudonyms to preserve their anonymity from other participants.

#### World Café

Considering the workloads of shelter workers and the challenges faced during the focus groups despite efforts to ensure accessibility (e.g., low participation, lack of access to a device for Telegram, lack of understanding of the technology), a World Café conversational process was held to deepen the results from the focus groups. The World Café process is a popular participatory method, increasingly used to collect qualitative data, and is well-suited to complement other research methods and validate findings (Löhr et al., 2020). This method uses a structured yet conversational approach, where predetermined topics are discussed in large, heterogeneous groups over successive rounds of conversation, with each round building on the previous one as the discussion progresses to subsequent participants (Löhr et al., 2020). The World Café conversation took place in a hotel conference room during our partner organization's annual meeting in May 2023, with a total participation of 161 shelter advocates (e.g., shelter workers, board members, coordinators, and directors). First, a summary of the focus group results was presented. Next, participants were randomly assigned to discuss a question in an initial 20-min round table session with an average of 10 participants per group. A total of six different questions were addressed during the World Café session, covering inquiries related to the four main categories that emerged from the asynchronous online focus groups. Additionally, two supplementary questions were incorporated so as to delve more into participants’ needs regarding intersectional feminist intervention approaches. After this topic had been briefly discussed, we gathered participants’ recommendations for the development of a training module on IPSV tailored for shelter workers. After the first round, participants were invited to engage in a second 20-min round, being randomly assigned to another question. This round began with a summary of a previous discussion that occurred during the initial round. Each table had a research assistant present to facilitate the discussion, take notes, and record the exchanges; this research assistant also summarized the key points addressed during the first 20-min round.

Prior to their participation, an information and consent form briefed participants on the procedures, benefits, and risks of their participation; sociodemographic questionnaires were also completed by the shelter advocates to collect descriptive data before the session took place. All participants gave their informed consent before participating. To thank participants for taking part in the study, eight $50 CAD gift cards were randomly awarded. This study was approved by the research ethics review committee of the authors’ affiliated university.

### Participants

The final sample consisted of 179 shelter advocates. Table 1 presents the sample characteristics of participants from each data collection method. In general, slightly more participants were in the 18–29 age bracket than in other age groups. A higher proportion of participants offered in-house counseling and had less than 3 years of experience, although participants with over 7 years of experience were almost equally represented in the World Café session. Participants came from all across Quebec (Canada), but mostly from rural areas.

**Table 1.** Sample Characteristics.

	Focus group participants(*n* = 18)		World Café participants(*n* = 161)	
Characteristics	*n*	%	*n*	%
Age group				
18–29	9	50.0	40	24.8
30–39	7	38.9	37	23.0
40–49	1	5.6	38	23.6
50 or more	1	5.6	38	23.6
Canadian	17	94.4	131	81.4
Main service offered				
In-house counseling	11	61.1	57	35.4
Animation of prevention workshop	5	27.8	28	17.3
Follow-up support services	2	11.1	7	4.3
Management	0	0.0	43	26.7
Years of experience (intervention)				
0­–less than 3 years	9	50.0	61	37.9
3–less than 7 years	4	22.2	23	14.3
7 years or more	5	27.8	60	33.5
Workplace areas				
Metropolitan areas	7	38.9	57	35.5
Rural areas	11	61.1	96	59.5

*Note.* Eight participants completed the consent form but did not complete the questionnaires during the World Café (5%). Years of experience were identified only for shelter workers; no information was available for participants in management positions.

Most participants (*n*_focus group_ = 14 [77.8%]; *n*_world cafe_ = 104 [64.6%]) were trained in social sciences (e.g., specialized education, social work, psychology, sexology, criminology) and therefore had some training on psychosocial interventions. In terms of IPSV training, the majority of the participants (*n*_focus group_ = 14 [77.8%]; *n*_world cafe_ = 111 [68.9%]) did not receive training on IPSV in their current or past work setting. Of those who received such training, three out of four participants in the online focus groups indicated they received training less than 5 years ago. In our World Café session, half of participants indicated having received training less than 5 years ago (*n* = 17) and half reported having received training more than 5 years ago (*n* = 17). Most participants (*n*_focus group_ = 12 [66.7%]; *n*_world cafe_ = 111 [68.9%]) revealed that they sometimes consulted documents (i.e., written, video, or other) about IPSV; a few participants revealed they never consulted such documentation (*n*_focus group_ = 2 [11.1%]; *n*_world cafe_ = 14 [8.7%]).

### Analysis

A conventional content analysis was conducted to allow training needs to emerge directly from the data, providing a deeper and more nuanced understanding of the phenomena under study (Hsieh & Shannon, 2005). This analysis allows researchers to explore and identify patterns within data sets without relying on preexisting theoretical categories, making it particularly valuable for studies in emerging or understudied fields (Hsieh & Shannon, 2005). However, this analysis can be time-consuming, and there is a risk of subjectivity in coding, as researchers’ interpretations may influence the results (Renz et al., 2018). This highlights the importance of transparency in detailing the process of data collection and analysis. Analysis from the transcripts of the focus groups was independently performed by two authors prior to the World Café session. A thorough reading of the transcripts was first undertaken to gain an overview of the data. A coding procedure was then applied, wherein the transcripts were segmented into units of meaning (i.e., sets of sentences related to the same idea; Tesch, 1990) and labeled to gain insights into the key concepts that emerged from the data. Finally, categorization was conducted to organize and draw connections within the data. This involved grouping the data into distinct categories and subcategories, facilitating a comprehensive understanding of the phenomenon being studied (Tesch, 1990). The data analysis was carried out consecutively (i.e., method by method), wherein the same steps were applied to the transcribed verbatims from the World Café session to enhance the results in light of the discussions surrounding the initial findings. Incorporating data triangulation was aimed to further analyze and deepen the results based on the insights gained during the World Café session. Data triangulation (i.e., consistency in the findings across different data sources) enhanced the credibility of our findings (Noble & Smith, 2015). The results from the World Café session were integrated with the initial findings from our online focus group; no additional categories or subcategories emerged. The specific contributions from the World Café session are identified in the results. The confidentiality of all participants was maintained by data anonymization (i.e., removing personally identifiable information such as first names, city, or names of shelters). The analysis was supported by *NVivo12 software*.

## Results

This study aimed to identify the training needs of shelter workers related to IPSV. Four primary needs emerged: (a) enhancing ease to address IPSV; (b) defining the scope and limits of interventions with IPSV survivors; (c) acquiring the skills needed to deal with IPSV; (d) guiding women in the medico-legal and legal procedures following IPSV disclosure. The needs are supported by participants’ own words during the sessions, with shelter workers’ years of experience bracket specified; however, the length of service is not available for those in management positions. An overview of the results is presented in Table 2.

**Table 2.** Overview of the Categories and Subcategories.

Categories	Subcategories
1. Enhancing ease to address IPSV	1.1 Navigating service users’ discomfort in addressing IPSV
	1.2. Managing their own discomfort related to sexuality and IPSV
	1.3 Eradicating the culture of silence in shelters to cultivate a supportive environment for IPSV interventions
2. Defining the scope and limits of interventions with IPSV survivors	2.1 Clarifying their mandate for IPSV-related interventions
	2.2 Identifying the limits of their interventions and knowing when to refer
3. Acquiring the skills needed to deal with IPSV	3.1 Becoming familiar with the best practices for dealing with IPSV
	3.2 Having concrete tools to discuss IPSV with women
4. Guiding women in the medico-legal and legal procedures following IPSV disclosure	4.1 Learning about the medico-legal process
	4.2 Mitigating the potential for contamination in the post-IPSV legal process
	4.3 Minimizing retraumatization in the medicolegal and judicial processes

### Enhancing Ease to Address IPSV

The discomfort surrounding IPSV emerged as a significant challenge. There is a crucial need to improve shelter workers’ comfort in engaging in IPSV discussions by addressing both service users’ discomfort and shelter worker's discomfort, as well as the culture of silence in shelters.

#### Navigating Service Users’ Discomfort in Addressing IPSV

##### Unveiling the unspoken: Navigating emotional barriers to IPSV disclosure in shelters

Participants emphasized that women seeking shelter for intimate partner violence often don’t initially report IPSV, but may gradually disclose experiences of IPSV after receiving support: “It is quite common that during their stay at the shelter, as they take the time to process their experiences, women reveal incidents of sexual violence” (P1—less than 3 years). Participants noted that the disclosure process can take time, as women may initially be reluctant to open up due to emotional challenges and internalized blame from their abusive partners: “Victims of violence internalize the blaming and shaming discourse from their aggressor. They experience shame, self-doubt, and a sense of responsibility for the violence they endure on a daily basis” (P2—7 years or more). Shelter workers needed the knowledge and skills to overcome emotional barriers and create a safe space for IPSV disclosure, with an emphasis on reducing shame and shifting blame from the victims to the perpetrators.

##### Acknowledging the unrecognized: Unraveling the layers of sexual consent and IPSV

Participants expressed the need for a deeper understanding of sexual consent and IPSV to address women's discomfort. They noted that survivors’ lack of understanding in this regard leads to unrecognized IPSV behaviors and discomfort. Societal norms which suggest that women “cannot be sexually assaulted by a partner and must meet their partner's sexual needs” (P3—7 years or more), coupled with media that reinforce these stereotypes and downplay more subtler forms such as sexual coercion, hinder women's recognition of IPSV. Shelter advocates stressed the importance of challenging these stereotypes and promoting a broader understanding of sexual consent and IPSV, including its existence in intimate relationships and its more subtle manifestations.

#### Managing Their Own Discomfort Related to Sexuality and IPSV

##### Sharing discomfort: Sexuality and IPSV as uneasy subjects to address from both sides

Participants revealed that many shelter workers also face their own discomfort when addressing sexuality or IPSV. Most World Café participants said that this was not a problem for them, but they acknowledged that their colleagues may face such challenges. They expressed that such discomfort primarily stems from the women's reluctance to discuss sexuality or IPSV: “When women talk to me about these subjects, I experience a slight discomfort, mainly concerning whether they feel comfortable discussing it with me” (P4—3–7 years).

Participants discussed the challenges of addressing sexuality due to its sensitive nature: “I find it challenging because of the taboo surrounding sexual issues in general. Sexuality is part of the domain of intimacy and privacy” (P5—7 years or more). World Café participants noted that an age gap between shelter service seekers and service providers may contribute to feelings of uncomfortableness, as discussing sexuality may feel more taboo among an older population. Participants also reflected on how the personal backgrounds of service providers, especially if they grew up in a conservative environment where “sexuality was considered taboo” or where they received limited sexuality education, could make them feel “less inclined to broach the subject” (P6—7 years or more). To address these challenges, participants emphasized the need to normalize discussions about sexual health, create an open atmosphere around sexuality in shelters, and include sexuality education in the training of shelter workers. This would enable them to question their own values regarding sexuality and improve their knowledge, confidence, and ability to address sexuality in their interventions.

Participants also discussed their own discomfort when receiving IPSV disclosures. They mentioned that many shelter workers have experienced sexual violence, which can lead them to draw parallels with their own experiences. Participants in the World Café session further elaborated on how this can lead to feelings of powerlessness. In order to effectively support survivors of IPSV, participants stressed the need for self-care and to “be able to be empathetic and not sympathetic” (P7—3–7 years), recognizing the importance of being compassionate without projecting one's own experiences onto service users. During the World Café session, the importance of adopting an intentional “intervener” posture was emphasized: “Being in the right posture makes it easier to receive stories of sexual violence. I aim to be in the position of listening, as an intervener, and not as the woman I am with my own values and experiences” (P8—3–7 years). Several participants recognized that new shelter workers find it difficult to adopt this posture and prioritize self-care because of a “strong desire to save everyone” (P9—7 years or more).

##### Setting boundaries: Leading by example

Participants mentioned the importance of establishing and maintaining personal boundaries when dealing with IPSV, with World Café participants stressing the need to respect these boundaries: “I tell the girls to listen to their limits. Do not venture into things you cannot handle, or you will end up feeling bad about it” (P10—management). They emphasized the need to recognize the strengths of team members and, when necessary, assign certain cases to colleagues who are better suited, such as referring IPSV cases to those with a background in sexology because “they have more tools to support them” (P11—7 years or more). Participants emphasized the importance of being role models for women and creating an environment where setting and respecting boundaries is both encouraged and normalized: “We say to women: ‘learn to set your boundaries, express your limits’. Set an example and say: ‘Look, here is my limit. I am sorry.’” (P12—7 years or more).

#### Eradicating the Culture of Silence in Shelters to Cultivate a Supportive Environment for IPSV Interventions

##### Breaking the silence: Fostering open conversations about IPSV among shelter workers

Participants pointed out the need for open discussions with their colleagues to share experiences and process emotions after interventions on IPSV: “After we focus on sexual assault, I need to take a break to refocus, review my personal values, and discuss how I feel with other workers” (P13—7 years or more). In some shelters, there is a culture of silence, and shelter workers avoid discussing their responses to IPSV. World Café participants expressed the importance of these conversations in alleviating discomfort: “It puts less pressure on me, because my team can help me with their perspective, that I may not have seen in the moment. It allows me to talk about it and helps me to intervene better.” (P14—3–7 years). One World Café participant shared how her colleagues’ discomfort affects her and leads to self-doubt: “When I sense that my colleagues are uncomfortable, it makes me question whether I should explore the topic with women. Their discomfort makes me question whether I lack the expertise or sensitivity to address it” (P15—3–7 years). Participants stressed the need for support from their colleagues and urged shelters to take steps to change their culture, to overcome discomfort around IPSV.

##### Building unity amid diversity: Managing tensions among women

World Café participants also highlighted the complex challenges of creating a cohesive environment. Tensions among service users were perceived to be exacerbated when addressing sexuality or IPSV, as women from different backgrounds (e.g., cultural) may hold different biases: “Everyone was against her because of her history of sexual exploitation. Sometimes it is not the shelter workers who will react and feel uncomfortable; but the other women” (P16—less than 3 years). In response, some expressed the need to break down biases about sexuality and IPSV through group interventions and to promote solidarity among women.

### Defining the Scope and Limits of Interventions With IPSV Survivors

Shelter workers reported a lack of clarity about their intervention mandate and the limits of their involvement in addressing IPSV, leading to questions about the scope of their support and the appropriate time to refer women to specialized sexual violence resources.

#### Clarifying Their Mandate for IPSV-related Interventions

##### Ensuring consistency: Capitalizing on commonalities in addressing intimate partner violence

Participants recognized that their role encompasses addressing all forms of intimate partner violence, including helping women recognize IPSV perpetrated by intimate partners or ex-partners: “It gives words to the discomfort she has felt in her relationship, a sense of not being valued, of being used, of being diminished” (P17—7 years or more). Although they felt confident in dealing with other forms of intimate partner violence, some found the guidelines for IPSV interventions less clear. Few participants emphasized that principles for other forms of intimate partner violence were applicable to IPSV. World Café participants felt relieved when they realized that they already “had it all” (P18—less than 3 years) in terms of the skills to effectively support IPSV survivors; this highlights their need for reassurance and confidence in their role.

#### Identifying the Limits of Their Interventions and Knowing When to Refer

##### Closing the gap: Navigating role ambiguity

Participants expressed challenges in determining the boundaries of their interventions with IPSV survivors. In particular, ambiguity stems from the lack of a clear distinction between their own mandate and that of a psychotherapist, and the fact that they do not want to interfere with the latter's role. As a result, many acknowledged their tendency to refer IPSV cases immediately, with some expressing discomfort with the speed of these referrals. During the World Café session, one director was outraged to learn that some shelters refer all IPSV cases: “They refer because they feel uncomfortable not knowing what to do, not feeling competent. By putting too much pressure on themselves, they end up doing nothing” (P19—management). This director discussed how IPSV survivors can fall into a gap between shelters and sexual violence services: “Shelters are experts in intimate partner violence, other resources are not uncomfortable with the issue of sexual violence, but they are uncomfortable with the domestic context” (P19—management).

##### Fostering cross-sector collaboration: Reducing referrals and service gaps

Participants mentioned instances where women faced barriers to accessing sexual violence services (e.g., lack of availability) or were reluctant to revisit their stories because of the trust bond already established with shelter workers. Some participants emphasized the importance of shelters collaborating with outside professionals or organizations (e.g., a resident physician) when women's needs are beyond their expertise in order to promote holistic recovery. They expressed the need for clear guidelines on when, how, and under what conditions to refer survivors to other resources in order to reduce the frequency of referrals and to avoid having women face challenges when seeking support. Referrals were mentioned in cases where women felt uncomfortable discussing IPSV or when the consequences of IPSV were prominent (e.g., sexual exploitation). During the World Café session, some participants mentioned referring women for long-term support, for sexual violence outside the domestic context (e.g., perpetrated by a father or brother), or in cases of revictimization.

### Acquiring the Skills Needed to Address IPSV

In order to feel prepared to address IPSV and foster an environment conducive to disclosure, participants emphasized effective intervention approaches and practical tools.

#### Becoming Familiar with the Best Practices for Dealing with IPSV

##### Unveiling the black box: Seeking to mitigate the risk of retraumatization

Participants expressed concern about triggering past traumatic experiences in IPSV survivors, with many stating that they discourage women from talking about IPSV: “We listen to women's experiences of intimate partner violence, but we stop them when it comes to sexual forms” (P20—3–7 years). They referred to IPSV as a “black box” that they should not delve into: “With sexual violence, we quickly express that it is not necessary to go into details. We use the black box technique to explain to women that by naming things, they may trigger memories” (P21—3–7 years). They expressed the need to acquire more knowledge about trauma-sensitive interventions tailored to IPSV survivors in order to reduce the risk of re-traumatization.

##### Fostering trauma-sensitive approaches: Understanding and responding to the impact of IPSV

Participants emphasized the need for further guidance on trauma-sensitive approaches. Given the stigma surrounding IPSV, they highlighted the need to build trust in order to create an environment in which women can disclose IPSV: “How to quickly build a strong sense of trust is particularly important to receive disclosures of sexual violence” (P22—7 years or more). Participants expressed a desire to learn more about best practices and common pitfalls, noting the importance of understanding the consequences of IPSV for their interventions: “Some may experience consequences that lead to fear of any form of touch or contact, including a comforting hand during intense emotions” (P23—7 years or more). World Café participants stressed the value of focusing on women's feelings rather than on the abuse, and valued training through modeling (e.g., role-playing to practice IPSV interventions, empathy, and nonjudgment).

##### Recognizing individuality: Tailoring support to survivors’ backgrounds

Participants highlighted the need to tailor support by considering the individual needs, pace, and beliefs that shape responses to IPSV. They noted how women who face multiple forms of oppression may be at higher risk for IPSV, may have different needs and beliefs, and encounter additional barriers when seeking support: “The oppression experienced (disability, illness, ethnicity, isolation, financial dependence, immigration) may result in fewer options, more barriers, and a longer tolerance of violence” (P24—7 years or more). Participants noted that immigrant women face specific forms of IPSV (e.g., being forced into arranged marriages) and are reluctant to talk about it, especially in group settings: “Many immigrant women are not comfortable talking about it in a group. Even when it comes to professional help, they usually say they don’t need or want it” (P25—7 years or more). Moreover, participants also emphasized the need to challenge beliefs such as “conjugal duty,” that shape women's roles in relationships: “For many, it is unthinkable to refuse sex to their aggressors because of religious or cultural beliefs. It goes deeply against their personal beliefs” (P26—7 years or more). Past experiences of trauma among First Nations people were also identified as contributing to the trivialization of IPSV: “One First Nations woman mentioned that she had experienced so many sexual assaults by different men that it had become normal for her to experience them” (P27—less than 3 years). Therefore, participants highlighted the need for training on IPSV-related beliefs and the experiences of women facing multiple oppressions. World Café participants reiterated the importance of culturally sensitive interventions, expressing concern about unintentionally sounding racist during interventions and emphasizing the need to be aware of one's biases. They suggested creating a glossary of cultural beliefs related to IPSV, and strategies to address them.

##### Privacy matters: Navigating IPSV disclosures in shared spaces

Participants raised questions about protecting women's privacy and confidentiality, noting that disclosures sometimes happen in shared spaces during workshops or in common areas (e.g., the kitchen). They needed guidance on how to handle IPSV disclosures in these instances: “When a woman talks about it in front of everyone, she is imposing her trauma. How do you deal with that without traumatizing others?” (P28—less than 3 years). World café participants valued informal discussions to address IPSV (e.g., broader discussions about sexual violence, misogyny, or gender norms) while stressing the importance of privacy. They also needed clarity on information sharing among colleagues, leading to recommendations of asking directly about women's comfort levels and preferences regarding the information that can be shared.

#### Having Concrete Tools to Discuss IPSV with Women

##### Equipping shelter workers: Providing specialized tools to address IPSV

Some participants mentioned a lack of resources specifically designed to address IPSV. They mentioned the need for simplified tools to help women recognize various manifestations of sexual coercion and violence, going beyond simple yes/no questions: “Shelter workers will often stop at the spontaneous no that women will say when asked if they have experienced IPSV, without digging deeper” (P29—7 years or more). Participants advocated for a structured approach (e.g., predefined questions and ready-to-use phrases) to build shelter workers’ confidence. World Café participants also highlighted the importance of practical, concrete, and easy-to-use tools, avoiding excessive theoretical complexity that may hinder real-life application.

### Guiding Women in the Medico-Legal and Legal Procedures Following a Disclosure of IPSV

Providing support to survivors of IPSV can be challenging when shelter workers lack familiarity with the medico-legal and legal procedures, as well as how to intervene without jeopardizing the integrity of legal claims and preventing the system from retraumatizing women.

#### Learning About the Medico-Legal Process

##### Filling the knowledge gap: Understanding the medico-legal process

Some participants expressed a lack of knowledge about the medico-legal kit, a medical examination designed to collect samples from sexual assault victims: “I was the first to suggest she go to the hospital following two assaults, the last one six days earlier. It was too late for the kit. I learned this while accompanying her” (P30—less than 3 years). Similarly, World Café participants revealed a limited understanding of the procedures, evidence collected, time constraints, and the professionals involved. They also discussed the perceived benefits of improved knowledge of the process, such as increased confidence in providing support. In turn, participants believed that women would feel more comfortable during the process and be better prepared to make informed decisions. Participants needed to understand how to support women before and after the process: “How do we prepare them to not be destabilized by this coldness?” (P31—7 years or more).

#### Mitigating the Potential for Contamination in the Post-ISPV Legal Process

##### Safeguarding women's rights: Navigating the tricky terrain of IPSV discussions and legal implications

Participants expressed reluctance to discuss IPSV due to concerns of the potential impact on legal proceedings if women choose to file complaints. They mentioned concerns about influencing women's complaints through their interventions: “I am afraid of contaminating through comments or questions that I might ask” (P32—less than 3 years). Participants revealed a need for clearer guidance on how to manage these conversations effectively without compromising women's rights and interests in seeking justice.

#### Minimizing Retraumatization in the Medico-Legal and Judicial Processes

##### Empowering women through the system: Addressing retraumatization and advocating for rights

Participants highlighted the potential for retraumatization within the medico-legal and legal processes, where women may be required to recount their experiences in detail, face intrusive questioning and cross-examination, or confront their perpetrators. World Café participants shared their experiences of witnessing retraumatizing situations: “One woman said that she was forced to perform oral sex, did not like it, and cried. The police officers said: “You finished it? So you consented?” Because she did it, they blamed her” (P33—7 years or more). Participants highlighted the need to be prepared to correct such harmful narratives among professionals (e.g., equating compliance with sexual consent), which can perpetuate victim-blaming attitudes and exacerbate the trauma experienced by IPSV survivors. World Café participants went a step further and offered some advice on how to intervene in these procedures. They stressed the need to accept that they cannot know everything, and to be transparent about it: “It can be scary, but being transparent and acknowledging that we will learn together. We will both grow. It is in line with our woman-to-woman approach” (P34—less than 3 years). They conveyed the need to advocate for women's rights in order to address any mistreatment or discrimination that women may encounter: “A physician was unpleasant, disrespectful, and judgmental. I complained to the hospital. We have to stand up for women's rights” (P35—7 years or more). Finally, they highlighted the importance of encouraging women to focus on the opportunity for empowerment rather than on the outcome (e.g., recognition of culpability by the system): “It's hard and challenging, if she has doubts, they need to be acknowledged and respected. I make sure to share that going through this process helps to regain control and reclaim power, but it is difficult” (P36—7 years or more).

## Discussion

This study provides important insights into the training needed to better support female survivors of IPSV from the perspective of women's shelter advocates. One of the strengths of the study is its participatory and emancipatory approach, in which researchers and stakeholders worked together to initiate actions for social change. In addition, the World Café method allowed for a more comprehensive collection of perspectives from a large and diverse group of key shelter advocates. This included individuals from a broader range of age groups compared to the online focus group participants, and those who had not been consulted in the initial data collection method (e.g., board members, shelter directors). These factors contribute to bolstering the overall rigor and validity of the study's findings and recommendations.

A key observation underscores the significant emotional discomfort both women and shelter workers may experience when discussing sexuality or IPSV. To address the challenges associated with this discomfort, it is essential to break down prevailing societal barriers and redefine IPSV as a serious public health issue. This shift requires moving away from conventional narratives that treat gender-based violence as a private matter, a perspective still prevalent in the narratives of many individuals (Bolton et al., 2024). From a psychological perspective, the literature has highlighted various approaches to reducing discomfort related to sexuality. Professionals have been encouraged to assess the importance they place on addressing sexual issues, reflect on their current competencies using existing skills, and actively pursue continuing education (O'Mullan et al., 2021). In addition, engagement in mentoring and peer support provided valuable guidance, that helped professionals manage discomfort, gain practical insights, and build confidence to effectively address sex-related issues in their practice (O'Mullan et al., 2021). In addition, addressing IPSV can be emotionally taxing, especially for shelter workers who have also personally experienced sexual violence. In the context of very high reciprocity with those they serve, caregivers are at risk of compassion fatigue and eventual burnout (Voth Schrag et al., 2022), and the complex emotional distress associated with caring for survivors of IPSV is no exception. Thus, there is a critical need for proactive measures to mitigate risk rather than waiting for problems to escalate before providing care; also with a focus on ensuring that the foundational principles of feminist intervention based on alliance amidst shared female oppression are upheld.

Consistent with findings from another study, participants in our study underscored the central role of a feminist and supportive shelter culture in effectively addressing the challenges faced, including those that accompany IPSV survivors’ interventions (Merchant & Whiting, 2015). In light of our findings, establishing a supportive shelter culture includes nurturing the well-being of shelter workers, promoting open communication, and encouraging self-care and boundary-setting practices, while maintaining a supportive alliance with service users, which may involve self-disclosure. It recognizes the demanding nature of the work undertaken by shelter workers and provides them with the resources and support they need to manage the emotional burden associated with IPSV interventions, thereby fostering support for service users.

Issues related to intersectional feminism are pervasive in the challenges faced by shelter workers, emphasizing the crucial importance of foregrounding this approach in interventions. Although shelter advocates characterize their practices as feminist, intersectional, and client-centered (Nichols, 2013), challenges persist in interventions related to IPSV. The overrepresentation of immigrant women and First Nations women in intimate partner violence shelters, coupled with Quebec being the Canadian province having the lowest proportion of agencies offering culturally diverse services (Moreau, 2019), underscores the need for services to be trained to provide culturally sensitive interventions. Our study participants highlighted that the impact of gender stereotypes, rape myths, and sexual violence is particularly pronounced in these populations. Furthermore, previous research has reported instances of racialized women experiencing racism from other women residing in shelters and sometimes from shelter advocates (Nnawulezi & Sullivan, 2014). Although the participants in our study did not report making racist comments, they explicitly expressed concerns about inadvertently sounding racist in their interventions. To address these challenges, it is essential to acknowledge the intersectionality of oppressions that women face and to recognize that cultural competency (i.e., awareness of one's own cultural biases and responses, and skills in providing culturally appropriate support) is not merely an option but a fundamental requirement for effective and inclusive support services (Brottman et al., 2020). In doing so, interventions can be better aligned with the principles of intersectional feminism, ensuring that the complexities of multiple identities and experiences are recognized and fully addressed.

Another need expressed by shelter advocates is to break down the compartmentalization of intimate partner violence and sexual violence in the development of policies aimed at tackling gender-based violence. This finding is consistent with those of previous studies revealing the need to break down this compartmentalization (Hattery, 2022; Helps, 2024; Webermann & Murphy, 2022). Often considered separately, intimate partner violence and sexual violence operate in silos within policy frameworks and service provision. Our findings show that survivors of IPSV face gaps in services, often having to recount their traumatic experiences and being shunted from one resource to another, with no integration between the two types of services. This siloed structure of community organizations in Canada and elsewhere can ultimately affect women's well-being, as repeating their stories can be counterproductive and cause re-traumatization. There is an urgent need to integrate a trauma-sensitive approach into all prevention practices for survivors, whether they have survived IPSV or any other form of gender-based violence (Raja et al., 2021).

### Limitations and Future Studies

While the World Café session provided valuable insights and encouraged open dialogue, it also had certain limitations. First, due to the limited time available for the in-person World Café, shelter advocates did not have the opportunity to express their views on all the issues identified in the online focus groups. In fact, each group was assigned to discuss only two out of eight topics. Secondly, given that shelter advocates are mostly professionals in the field of intimate partner violence, it might be difficult for some of them to admit that they might have difficulties dealing with IPSV, especially during the World Café session, where they were not anonymous and the group was heterogeneous (shelter worker, directors, people with more and less experience). The desire to present themselves in a positive light to peers and colleagues may have prevented participants from being fully vulnerable and expressing their true feelings and perspectives. This limitation may have been exacerbated by the presentation of a summary of the focus group results at the outset, which may have made participants feel pressured to agree with the summary or frame their discussions around it, rather than explore alternative or novel ideas. As a result, important perspectives or insights may not have been fully explored or shared during the World Café session, and this should be taken into account when interpreting the results. In addition, it was not possible to match the online focus group participants with those in the in-person World Café session. Therefore, some participants may have taken part in both, which may result in a smaller final sample size than indicated. Finally, there were limitations to the focus groups. Potential selection biases in the recruitment of shelter workers, such as the overrepresentation of those who are particularly motivated by the topic, more comfortable discussing sexuality, and more familiar with or involved in IPSV interventions, may result in findings that do not fully reflect the experiences or perspectives of all shelter workers. The asynchronous nature of the online discussions may have led to less detailed responses from participants compared to face-to-face interactions. The lack of immediate feedback or real-time dialogue likely limited opportunities for deeper conversations and for participants to respond to and expand on points raised by others. In face-to-face settings, the flow of conversation tends to be more dynamic, allowing for a more organic exchange where participants can build on each other's ideas, ask clarifying questions, and engage more interactively. Some of the less tech-savvy participants did not take part because they were uncomfortable with the technology or had difficulty installing the chat application. Future studies could therefore adapt the data collection to ensure anonymity, while still considering those who are less skilled with technology in conducting individual interviews.

Shelter advocates spoke of their perceptions of the discomfort of service users around sexuality and IPSV, which may impact their own comfort. Future studies could probe women in shelters who have experienced IPSV about the help they received and their experiences with shelter workers (e.g., in relation to medico-legal aspects, their service trajectories, attention to trauma-informed care, and recognition of intersectionality). In addition, future studies could also investigate the ways in which women would have liked to have been supported in such cases. Cross-referencing data could complement the training needs of shelter workers. Giving the urgent need for training, future research should focus on developing and evaluating a training program targeting IPSV for shelter workers.

### Implications for Practice

A better understanding of the challenges and needs related to IPSV that shelter workers face in their practice with female survivors of intimate partner violence enables better support for service users. At the intersection of intimate partner violence and sexual violence, IPSV is an overlooked topic in the training currently provided to shelter workers. Our study highlights numerous knowledge, attitudes, and skills that shelter workers need to acquire through targeted training programs to equip them to support female survivors of IPSV. Such an initiative is particularly innovative because it directly addresses a pressing need, clearly identified by shelter workers. This initiative is an important step towards more appropriate, effective, and sensitive interventions for female survivors of IPSV. Our findings support the need to educate shelter workers about sexual violence in order to better support women. Training should present the continuum of IPSV, ranging from attitudes and beliefs that normalize IPSV to physical forms of sexual violence (e.g., https://www.ualberta.ca/current-students/sexual-assault-centre/create-change.html). It should also address the myths associated with sexual consent and IPSV, such as that one cannot be sexually assaulted by one's partner, or that once consent has been given for sexual activity, consent will prevail for future sexual activities. By knowing more about the forms of IPSV and the continuum of coercion and violence, they will be better able to recognize them and deconstruct certain myths held by service users. In terms of knowledge, given the need to better understand the medico-legal process, training for shelter workers should address this content. Demystifying the process and timelines for the medico-legal kit, the complaint process and elements of the nonsuggestive interview could help shelter workers to better accompany female survivors of IPSV.

Also, given that some shelter workers express a lack of comfort in discussing IPSV with service users, a training program for shelter workers should encourage them to reflect on and discuss their own values related to sexual health (e.g., consent, communication, pleasure). Reflecting on and discussing these matters helps to clarify their values and boundaries, and may help shelter workers feel more comfortable talking freely about these issues.

Given that shelter workers express a lack of confidence in their ability to discuss IPSV, training offerings should provide concrete examples of interventions based on best practices. For example, experienced shelter workers could use role-playing to demonstrate best practices when receiving an IPSV disclosure or when responding to a woman's comment about another service user's experience of IPSV. Modeling is a proven pedagogical strategy for building confidence in one's own abilities (Bandura, 1977). Another strategy for building confidence in one's abilities is verbal persuasion, i.e., having credible people, such as work colleagues, who point out the good work of others and insist that they can make relevant and effective interventions (Bandura, 1977). In a training context, this strategy could be used to make shelter workers aware that they have the resources and skills to intervene with women because they are already doing so with other forms of intimate partner violence.

Furthermore, given the diverse backgrounds of service users in terms of culture and traumatic experiences, it would be beneficial for training to further explore trauma-informed care. While shelter workers already receive some training in this area, the findings indicate a need for a deeper understanding, particularly regarding effective interventions for IPSV survivors in alignment with trauma-informed principles. This approach takes into account past trauma and its effects, with the goal of designing integrative interventions that prevent retraumatization (Elliott et al., 2005). Trauma-informed care can help build trust between service providers and survivors while emphasizing women's empowerment, among other benefits. The intersectional approach could also be relevant to learning how to deal with values different from one's own in relation to sexuality and IPSV.

Our findings suggest that effective advocacy begins with personal growth and self-awareness. Shelter workers must prioritize their own well-being and recovery before they can help other women recover. Training should focus on the importance of developing, within the shelter, a culture of support and sharing among team members. Concretely, this could take the form of group supervision, where shelter workers can share their experiences of IPSV and the challenges they face. It is crucial to address this burden by providing training, giving them access to resources, keeping their mental health in mind throughout their mandate, and fostering a culture of support within shelters. It is essential that staff are trained and encouraged to work on themselves before they can help other women. Workers such as those in intimate partner violence shelters who work with traumatized service users are at risk of compassion fatigue and vicarious trauma (Kim et al., 2022; Voth Schrag et al., 2022). One effective way to prevent these consequences from constant exposure to the suffering and trauma of others is to implement peer debriefing sessions, which have been shown to be effective in reducing the risk of vicarious trauma (Scott et al., 2022).

This study represents an important step toward a more structured, effective intervention that is sensitive to the realities and traumas of women who are survivors of IPSV. Ultimately, shelter workers who feel more confident in their interventions could lead to a significant improvement in the quality of services offered to women who are survivors of IPSV and to fewer referrals. As a result, women who use these services should feel better supported and accompanied, thus promoting their recovery, and reducing the risk of sexual and intimate revictimization.
